# Protective Effect of Alkaline Mineral Water on Calcium Oxalate-Induced Kidney Injury in Mice

**DOI:** 10.1155/2023/4559802

**Published:** 2023-10-25

**Authors:** Lei Liu, Chen Lin, Xiu Li, Yulan Cheng, Rui Wang, Chao Luo, Xinyuan Zhao, Zhitao Jiang

**Affiliations:** ^1^Department of Pathology, Affiliated Hospital of Nantong University, Nantong, China; ^2^Vectors and Parasitosis Control and Prevention Section, Center of Disease Prevention and Control in Pudong New Area of Shanghai, Shanghai, China; ^3^School of Food Science and Technology, Jiangnan University, Wuxi, China; ^4^Department of Occupational Medicine and Environmental Toxicology, Nantong Key Laboratory of Environmental Toxicology, School of Public Health, Nantong University, Nantong, China; ^5^College of Mechanical and Electrical Engineering, China Jiliang University, Hangzhou, China

## Abstract

**Background:**

Kidney stone disease induces chronic renal insufficiency by crystal-induced renal tubular epithelial cell injury. It has been reported that the prevalence of kidney stone disease is increasing, accompanied by the high recurrence rate. Alkaline mineral water has been reported to possess beneficial effects to attenuate inflammation. Here, we explored the potential protective effects and underlying mechanisms of alkaline mineral water against calcium oxalate-induced kidney injury.

**Methods:**

We performed the mice kidney stone model by administering glyoxylate at 100 mg/kg once daily for 7 days. To assess the effects of alkaline mineral water on oxalate-induced kidney injury, mice drank different water (distilled water, natural mineral water at pH = 8.0, as well as natural mineral water at pH = 9.3) for 7 days, respectively, followed by glyoxylate exposure. After collection, crystal formation, kidney injury and cell apoptosis, fibrosis, oxidative stress, as well as inflammation were measured.

**Results:**

Our results showed that glyoxylate treatment led to kidney crystal formation and fibrosis, which can be attenuated by drinking alkaline mineral water. Furthermore, alkaline mineral water also reduced kidney injury and cell apoptosis, oxidative stress, and inflammation.

**Conclusion:**

Alkaline mineral water supplement prevents progression of glyoxylate-induced kidney stones through alleviating oxidative stress and inflammation.

## 1. Introduction

Nephrolithiasis (kidney stones) is one of the most prevalent urologic diseases worldwide, with a lifetime occurrence over 10% in the United States [1] and 6.4% in China [2] according to the recent data. Research has found that the formation of kidney stone is due to the imbalance of inhibitors and promoters of crystallization [3]. The characteristic symptoms of kidney stones are cramping and intermittent abdominal and flank pain, as well as hematuria, nausea or vomiting, and malaise, which impart substantial long-term disease burden. There are growing data for an increasing incidence and recurrence of kidney stones in all age, sex, and racial and/or ethnic subgroups, posing a serious threat to human health.

Understanding of pathophysiology of kidney stones is important to develop efficient strategies of prevention and treatment. Globally, approximately 80–90% of all kidney stones are composed of calcium oxalate (CaOx) mixed with calcium phosphate or uric acid [4, 5]. CaOx forms, grows, aggregates, and finally retains within the kidneys due to urinary supersaturation [6]. CaOx crystals induce intrarenal inflammation and kidney tubular cell injury, which is strongly associated with oxidative stress injury and reactive oxygen species (ROS) [7, 8]. Inhibition of renal inflammation and ROS production has been identified to alleviate oxidative stress damage and reduce intrarenal crystal deposition [9].

The risk factors contributing to kidney stones are various, including renal anatomic abnormalities, family history, older age, metabolic syndrome, climate changes, lifestyle, microbiomes, and so on [10–14]. In practice, lifestyle interventions are shown effective and economic from all available treatment options. People have been trying to find the commonly espoused nonprescription agents or dietary recommendations to prevent stone formation [15]. Recently, compelling links are beginning to emerge between high and appropriate fluid intake and reduced stone recurrence [16]. The quality of drinking water, including the hydrogen bond network [17], high pH [18], disturbed ratio of Ca and Mg [19], high salt [20], increased intake [21], and even specific beverage types, is believed to be conducive to the formation of kidney stones, offering drinking behavior along with dietary modification designed to restore normal renal biochemistry.

Despite these advances, the inescapable elephant in the room remains about treatment and prevention of kidney stones. Here, we focused on the pH of drinking water and explored the preventive and protective roles of alkaline drinking water (pH = 9.3) on the development of kidney stones. We found that a high pH drinking water reduced calcium stones' deposition in renal tubular epithelial cells, which further uncovering the underlying mechanism involved in kidney stones' formation.

## 2. Materials and Methods

### 2.1. Reagents and Antibodies

The glyoxylate was provided by Sigma (G4502). GSH (A006-1-1) and MDA (A003-1-2) were from Nanjing Jiancheng Bioengineering Institute. The SOD (E-BC-K020-M) was purchased from Elabscience. The primary antibodies against OPN (22925), CD44 (15675), Nrf2 (16396), HO-1 (10701), and SOD-1 (10269) were purchased from Proteintech Company. Cell Signaling Technology provided primary antibodies against c-c3 (cleaved-caspase-3) (96611) and SIRT1 (8469). Primary antibodies against *α*-SMA (CY5295) were bought from Abways, and MCP-1 (DF7577) was from Affinity. Alkaline natural mineral water at high pH (pH 9.3, Shilin Tianwaitian) and alkaline natural mineral water at low pH (pH 8.3, Shilin Tianwaitian) were purchased from Jingdong Online Mall.

### 2.2. Mouse Model of Kidney Stones and Treatment

C57BL mice (6–8-weeks old) were provided by the Experimental Animal Research Center of Jiangnan University and raised in the Animal Facilities of Jiangnan University under pathogen-free conditions. All experimental procedures followed the rules of the National Institutes of Health Guide for the Care and Use of Laboratory Animals. The mice were divided into the following 4 groups: (1) the control group (drinking distilled water without glyoxylate treatment), (2) the model group (glyoxylate-induced kidney stones group with drinking distilled water), (3) the model + low pH group (glyoxylate-induced kidney stones group with natural mineral water at pH = 8.3), and (4) the model + high pH group (glyoxylate-induced kidney stones group with natural mineral water at pH = 9.3). Before glyoxylate exposure, the mice drank corresponding water for 1 week (1–7 day). To create the glyoxylate-induced kidney stones model, each mouse received either intraperitoneal vehicle (saline) or glyoxylate (glyoxylic acid, GA) (100 mg/kg, 100 *μ*l) once daily on day of 8–14. During the abovementioned period, we recorded the body weight and water consumption (Table S1 and Table S2). Finally, the left kidney was frozen at −80°C for future use, and the right kidney was fixed in 4% paraformaldehyde for histologic examination.

### 2.3. Histological Analysis

The kidney tissues were fixed in 4% formalin and paraffin-embedded, followed by sectioned at 4 *μ*m. After being deparaffinized and rehydrated, the prepared slices were used to conduct pathological staining according to the established standard procedure.

For hematoxylin and eosin (HE) staining, slices were immersed in hematoxylin for 10 min and eosin for 2 min.

Masson trichrome staining, for determining the levels of collagen deposition, was performed by the manufacturer's instruction. After the slices were dyed by Weigert's iron hematoxylin solution and washed by distilled water for 3 times, the slices were sequentially stained by 0.7% Masson‐Ponceau‐acid fuchsin staining solution for 10 min, differentiated in phosphomolybdic acid for 4 min, and then stained by 2% aniline blue dye solution. Finally, the collagen deposition was observed under a light microscope.

Periodic acid-Schiff (PAS) staining was performed as follows. The periodate oxidation solution was firstly added on the kidney sections for 5 min, and the Schiff reagent was stopped until the color of the tissue changed to red-purple. The results were observed after hematoxylin staining.

Sirius red staining was conducted according to the manufacturer's instruction with commercial kits. The positive staining was shown after the kidney sections were stained with Sirius red dye for 1 h.

Von Kossa staining commercial kit was applied to detect the calcium salt in the kidneys. Briefly, silver nitrate and hematoxylin and eosin sequentially immersed the tissues, and the staining was finished.

### 2.4. Tunel Assay

In situ cell death detection kit was used to perform the Tunel assay to determine the apoptotic cells in kidney tissue. Briefly, the slices were placed in a humidified/dark chamber and incubated with the Tunel reaction mixture for 1 h at 37°C following deparaffinized and permeabilized by 0.1 M sodium citrate, pH 6.0 at 65°C for 30 min. After that, we observed the positive nuclear staining under the fluorescence microscope.

### 2.5. Reverse Transcription-Polymerase Chain Reaction (RT-PCR)

The detailed procedure was conducted as our previous description [22]. Total RNAs were extracted from mouse kidney samples by the Trizol reagent. Then, we measured RNA concentrations using a spectrophotometer (One Drop, OD-1000+). Next, extracted RNAs were reverse transcribed with the Omniscript RT-PCR kit (Qiagen, Germany), and amplified products by specific primers (Sangon, Shanghai) were quantified following the manufacturer's protocol. Primer sequences are provided in Table 1. The PCR products were analyzed by electrophoresis using 2% agarose gels, and the density of the bands was used to quantify the mRNA using glyceraldehyde 3-phosphate dehydrogenase (GAPDH) mRNA as an internal control.

### 2.6. Statistical Analysis

All data are presented as the mean ± SD. Statistically significant differences between the control group and treated groups were determined by Students *t*-test or one-way analysis of variance (ANOVA) carried out with GraphPad Prism 5.0. To analyze the correlation between genes, Pearson's correlation test was applied. *P* values <0.05 were considered statistically significant.

## 3. Results

### 3.1. Alkaline Mineral Water Mitigated Glyoxylate-Induced Renal Crystal Formation and Fibrosis

**Figure 1:**
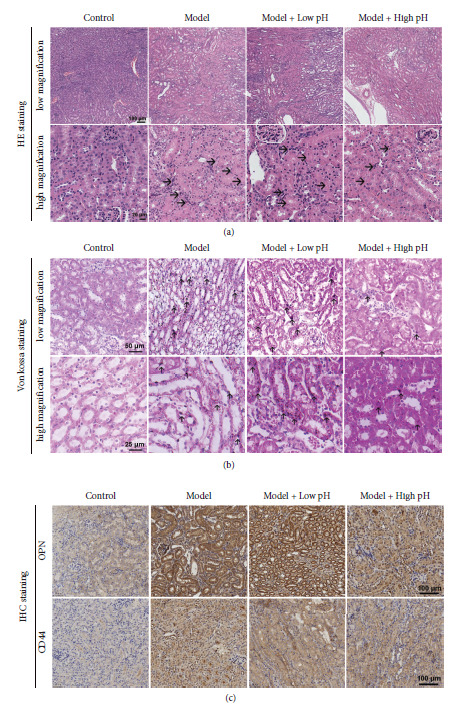
Effects of alkaline mineral water on renal CaOx crystal deposition and tubular injury in mice. (a, b) Representative images for the HE staining and Von Kossa staining of calcium deposition in mice kidneys. (c) OPN and CD44 immunohistochemistry staining of paraffin embedded kidney sections (scale bar = 100 *μ*m).

Mice were divided into 4 groups to determine the effect of alkaline mineral water supplement on the progression of kidney stones as described in materials' section. The analysis of HE and Von Kossa staining revealed a high degree of renal damage with dilated and ruptured tubules in the model group, indicating profound crystal deposition staining (Figures 1(a) and 1(b)). Von Kossa staining showed that CaOx crystals formed and deposited in the renal tubules between cortex and medulla in the model mice. High pH mineral water rather than low pH mineral water significantly reduced the number of crystals. Next, we assessed the protein expression levels of the osteopontin (OPN) and CD44 since they are crystal-related gene and crystal adhesion-related gene, respectively [23]. The results of IHC showed that a drastic increase of OPN and CD44 expression in the model group when compared with the control group (Figure 1(c)). Similarly, high pH mineral water significantly downregulated their expression upon CaOx stimulation (Figure 1(c)), while low pH mineral water supplement failed to change their expression levels (Figure 1(c)). Furthermore, we also found high pH mineral water effectively decreased the uric acid level compared with the model group, although urea nitrogen and creatinine failed to be reduced (Figure S1).

**Figure 2:**
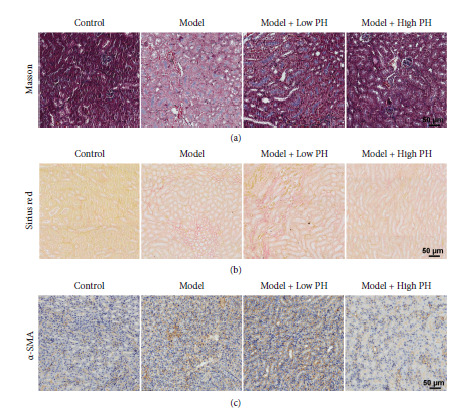
Alkaline mineral water inhibited glyoxylate-induced renal fibrosis in mice. Representative images showing Masson trichrome (a), sirius red (b), and *α*-SMA staining (c) by immunohistochemistry (scale bar = 50 *μ*m).

Tubulointerstitial fibrosis has been reported as a key pathophysiological process in oxalate nephropathy with renal failure [24]. To evaluate the alterations of fibrosis levels in glyoxylate-damaged renal tissue in four groups, we performed Masson staining and Sirius red staining. Masson staining of renal tissues showed overt fibrosis in the model group compared to the control group, which was significantly reversed after high pH mineral water supplement (Figure 2(a)). Reduced fibrosis was also revealed by Sirius red and *α*-SMA staining, which are both markers for deposition of extracellular matrix (ECM) (Figures 2(b) and 2(c)). Similar invalid effects were also found in the low pH mineral water supplement group (Figures 2(a)–2(c)). Taken together, these results indicated that high pH mineral water intake prevents glyoxylate-induced renal crystal formation and fibrosis.

### 3.2. Alkaline Mineral Water Attenuated Glyoxylate-Induced Renal Damage and Cell Apoptosis

**Figure 3:**
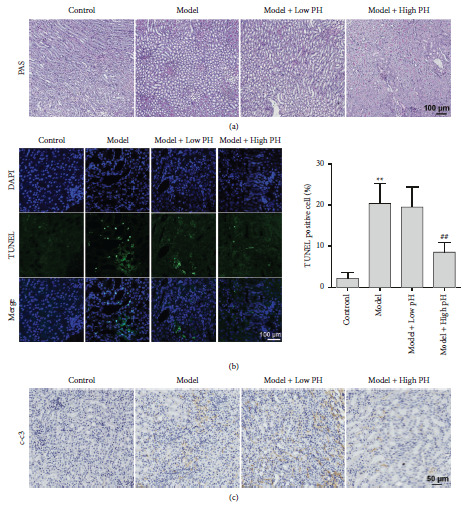
The effects of alkaline mineral water on glyoxylate-induced apoptosis in mice. (a) PAS staining of mice kidney from different groups. (b) Tunel staining was used to assess renal apoptosis (scale bar = 100 *μ*m). The percentages of tunel-positive cells were calculated and showed right. Data are presented as the mean ± SD. ^*∗∗*^*P* <  0.01 vs. the normal control group, ^##^*P*  <  0.01 vs. the model group. (c) The expression of apoptosis-related protein c-c3 (cleaved-caspase-3) was evaluated by immunohistochemistry (scale bar = 50 *μ*m).

It is well reported that apoptosis is involved in CaOx crystal formation and renal damage [25]. PAS staining revealed significant increase of tubular injury in model groups as showed by reduced PAS positively stained cells than the control group (Figure 3(a)). Supplement of high pH mineral water dramatically alleviated tubular injury in model mice (Figure 3(a)). In addition, the Tunel staining results showed that Tunel-positive cells dramatically increased in the model group compared with the control group, whereas impressively fewer apoptotic cells were detected in the high pH group rather than in the low pH group (Figure 3(b)). IHC results of cleaved caspase 3 further clarified the abovementioned results (Figure 3(c)). Collectively, these results suggested that pretreatment of high pH mineral water had a stronger ability to reduce apoptosis of renal cells than the low pH mineral water, therefore presenting anti-injury against CaOx-induced kidney damage.

### 3.3. Alkaline Mineral Water Alleviated Glyoxylate-Induced Renal Oxidative Stress

**Figure 4:**
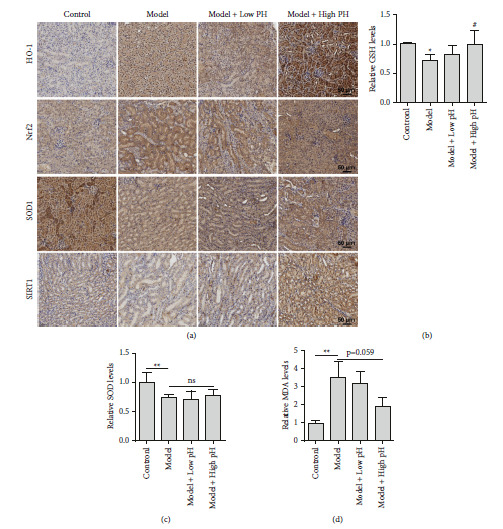
Alkaline mineral water inhibited glyoxylate-induced oxidative in mice. (a) HO-1, Nrf2, SOD-1, and SIRT1 expression assay using immunohistochemistry staining of paraffin embedded kidney sections (scale bar = 50 *μ*m). (b–d) Mice were treated as shown, and then relative GSH (b), SOD (c), MDA, and (d) content of kidney tissues were detected by their corresponding kits. All quantitative data are shown as means ± SD, ^*∗*^*P* <  0.05 vs. the normal control group, ^*∗∗*^*P* <  0.01 vs. the normal control group, ^#^*P* <  0.05 vs. the model group.

A good many of studies have showed that oxidative stress contributed to CaOx-induced crystal formation [26, 27]. Therefore, we investigated whether oxidative stress is participated in alkaline mineral water-induced therapeutic effects. Nrf2/HO-1 is a major antioxidant pathway involved in CaOx-induced crystal deposition treatment [23, 28]. We found that both Nrf2 and HO-1 were upregulated after CaOx treatment, and the upregulation was further magnified upon high pH mineral water supplement (Figure 4(a)). Furthermore, SOD downregulation contributed to CaOx-induced oxidative injury, although it was not affected by high pH mineral water supplement (Figure 4(a)). Recently, SIRT1 reduction was reported to participate in CaOx-induced crystal formation [29]. Interestingly, high pH mineral water supplement dramatically stimulated the SIRT1 expression level than the model group (Figure 4(a)). Additionally, GSH, MDA, and SOD levels were measured. Although there was no evident change in SOD levels when treated with alkaline mineral water compared to the model group, higher GSH levels were found in the model + high pH group (Figures 4(b) and 4(c)). Meanwhile, MDA levels showed a downward trend (Figure 4(d)). Based on the abovementioned findings, it was suggested that treatment of alkaline mineral water could ameliorate oxidative damage in glyoxylate-induced renal injury.

### 3.4. Alkaline Mineral Water Attenuated Glyoxylate-Induced Renal Inflammation

**Figure 5:**
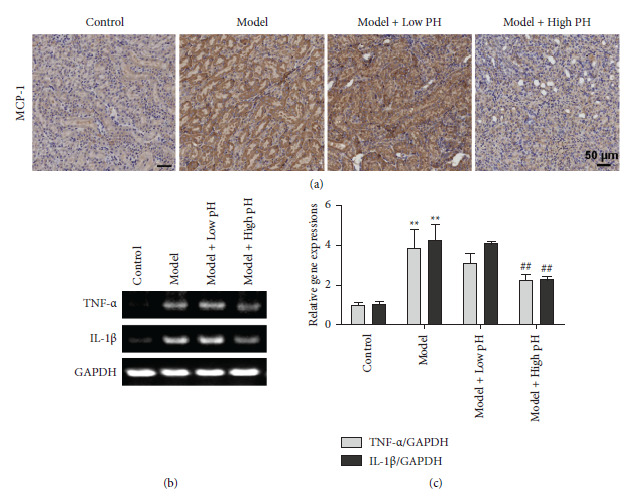
Alkaline mineral water alleviated glyoxylate-induced inflammation in mice. (a) MCP-1 expression assay using immunohistochemistry staining of paraffin embedded kidney sections (scale bar = 50 *μ*m). (b) Mice were treated as shown and then relative IL-1*β* and TNF-*α*mRNA expression levels were detected by RT-PCR. (c) Quantitative results of (b). All quantitative data are shown as means ± SD, ^*∗∗*^*P* <  0.01 vs. the normal control group, ^##^*P* <  0.01 vs. the model group.

Many investigations have indicated that calcium oxalate stone formation is dependent on the inflammatory process and secretes many inflammatory factors, such as monocyte chemotactic protein 1 (MCP-1) [30]. Therefore, we assessed the effects of alkaline mineral water on glyoxylate-induced renal inflammation through measuring MCP-1 expression. We found that renal MCP-1 was significantly higher in the model group than the control group, but the effect was reversed after high pH mineral water supplement (Figure 5(a)). Moreover, IL-1*β* and TNF-*α* mRNA expression levels were dramatically increased in kidney tissue from the model group, and their expressions further reduced after high pH mineral water supplement (Figures 5(b) and 5(c)). Based on the abovementioned results, it was suggested that alkaline mineral water could assuage glyoxylate-induced renal inflammation.

## 4. Discussion

CaOx-induced nephrolithiasis is one of the primary causes of chronic renal diseases, leading to heavy expenditure burden and poor life quality [31]. The current development of medical treatment mainly focuses on limiting oxalate intake and oxalic acid production [32]. However, dietary and drinking habits are believed to be economic, useful, and of good treatment compliance, with a minimal side effect possibility. In the present study, we focused on the protective effect of alkaline mineral water supplement and its potential mechanisms in the glyoxylate-induced kidney injury. Our results show that alkaline mineral water inhibited oxalate-induced crystal formation and deposition in vivo. Specifically, we found that alkaline mineral water at high pH had a protective effect on oxalate nephropathy by reducing renal fibrosis, apoptosis, oxidation, and inflammation. All data implied that alkaline mineral water may be an effective and preventive treatment for oxalate-induced nephrotoxicity.

Kidney crystal formation is a complicated process involved with oxidative stress, mitochondrial membrane potential, apoptosis, inflammatory response, etc. [33, 34]. Excessive oxidative stress can induce inflammation and cell damage, indicating that oxidative stress is one of the most important links in stone formation. Previous studies indicated that oxidative stress contributes to the pathogenesis of nephrolithiasis. While application of antioxidants exerts a therapeutic effect, long-term clinical trials are limited because some of antioxidant-related interventions are invalid or harmful [35]. These failures may come from exogenous antioxidants which incur inflammation at the same time [36, 37]. We here showed that alkaline water attenuated oxidative stress without inducing inflammation, which overcame the limitation.

Quality of water gas gained much attention for kidney stone formation. Water hardness [38, 39], calcium content [40], and beverage types [41, 42] have been recently reported to influence the incidence of kidney stones. However, controversy remains as to whether the pH of drinking water impacts the occurrence of stone formation [43]. Urine pH and related assessment provide essential information about stone formation potential that can guide prevention. Furthermore, several antinephrolithiasis drugs have been confirmed to increase urinary pH, thereby providing the possibility of stone dissolution [44]. So far, there is no distinguished evidence linking the pH of drinking water to that of urine. Since kidney stone formation is a multiple process composed of CaOx crystallization, crystal growth, aggregation, adhesion, and retention [45], alkaline mineral water has the potential against stone formation in every step.

## 5. Conclusion

In summary, we developed a model of glyoxylate-induced kidney stones by repetitive administration of glyoxylate. By using this model, we have demonstrated that mice fed with alkaline mineral water are partly protected from progressive renal impairment. We thus believe that alkaline water may have the potential and promising value for the treatment of CaOx nephrolithiasis.

## Figures and Tables

**Table 1:** DNA sequences of primers for polymerase chain reaction.

Gene name	Primer name	Sequence (5′-3′)
Mouse-GAPDH	GAPDH-F	TGAACGGGAAGCTCACTGG
	GAPDH-R	TCCACCACCCTGTTGCTGTA

Mouse- IL-1*β*	IL-1*β*-F	AGCAGCTATGGCAACTGTTC
	IL-1*β*-R	AATGAGTGATACTGCCTGCC

Mouse-TNF-*α*	TNF-*α*-F	ATGTCTCAGCCTCTTCTCATTC
	TNF-*α*-R	GCTTGTCACTCGAATTTTGAGA

## Data Availability

The datasets used and/or analyzed during the current study are available from the corresponding author on reasonable request.

## Abbreviations

ANOVA: Analysis of variance
CaOx: Calcium oxalate
c-c3: Cleaved-caspase-3
ECM: Extracellular matrix
GA: Glyoxylic acid
GAPDH: Glyceraldehyde 3-phosphate dehydrogenase
GSH: Glutathione
HE: Hematoxylin and eosin
IHC: Immunohistochemical
MCP-1: Monocyte chemotactic protein 1
MDA: Malonaldehyde
Nephrolithiasis: Kidney stones
OPN: Osteopontin
PAS: Periodic acid-Schiff
ROS: Reactive oxygen species
RT-PCR: Reverse transcription-polymerase chain reaction
SOD: superoxide dismutase
Tunel: Terminal deoxynucleotidyl transferase mediated nick end labeling.

## Supplementary Materials

Supplementary Materials Figure S1: the serum BUN (A), creatinine (B), and uric acid (C) levels in the model group and high pH group. Table S1: the body weight of mice after calcium oxalate exposure. Table S2: the water consumption of mice after calcium oxalate exposure.
