# Supplementary material for: Protective Effect of Alkaline Mineral Water on Calcium Oxalate-Induced Kidney Injury in Mice
Source: Evid Based Complement Alternat Med. 2023 Oct 25;2023:4559802. doi: 10.1155/2023/4559802 (PMC10620026; doi:10.1155/2023/4559802)
Supplement: Supplementary Materials — Figure S1: the serum BUN (A), creatinine (B), and uric acid (C) levels in the model group and high pH group. Table S1: the body weight of mice after calcium oxalate exposure. Table S2: the water consumption of mice after calcium oxalate exposure. [file 4559802.f1.doc]

**Protective effect of alkaline mineral water on calcium** **oxalate-induced kidney injury in mice**

Table S1: the body weight of mice after calcium oxalate exposure

|  | 0 day | 1 day | 2 day | 3 day | 4 day | 5 day | 6 day | 7 day |
| --- | --- | --- | --- | --- | --- | --- | --- | --- |
| Control | 23.50±  0.85 g | 23.72 ±  0.98 g | 23.81 ±  0.90 g | 23.84 ±  1.23 g | 23.89 ±  0.96 g | 23.63 ±  1.01 g | 24.10 ±  1.06 g | 23.11 ±  0.97 g |
| Model | 22.93 ±  0.82 g | 22.41 ±  1.16 g | 22.62 ±  1.14 g | 22.82 ±  1.18 g | 22.93 ±  1.27 g | 22.31 ±  1.21 g | 23.04 ±  1.29 g | 22.50 ±  1.01 g |
| Model + low pH | 22.41 ±  1.98 g | 22.70 ±  1.40 g | 22.48 ±  1.39 g | 23.05 ±  1.06 g | 23.06 ±  1.32 g | 23.05 ±  1.33 g | 23.26 ±  1.28 g | 22.50 ±  1.06 g |
| Model + high pH | 23.04 ±  0.90 g | 22.90 ±  1.15 g | 23.19 ±  1.12 g | 23.13 ±  0.97 g | 23.18 ±  1.05 g | 22.93 ±  1.36 g | 22.95 ±  1.37 g | 22.43 ±  1.72 g |

Table S2: the water consumption of mice after calcium oxalate exposure

|  | 0 day | 1 day | 2 day | 3 day | 4 day | 5 day | 6 day | 7 day |
| --- | --- | --- | --- | --- | --- | --- | --- | --- |
| Control | 4.44 ±  0.20 mL | 3.80 ±  0.27mL | 4.62 ±  0.35mL | 4.77 ±  0.98mL | 4.32 ±  1.14mL | 4.17 ±  0.82mL | 4.58 ±  0.75mL | 5.86 ±  2.09 mL |
| Model | 5.37 ±  0.26mL | 5.99 ±  0.54mL | 6.17 ±  0.89mL | 5.82 ±  0.72mL | 5.93 ±  1.27mL | 5.17 ±  0.33mL | 6.18 ±  0.88 mL | 6.62 ±  1.43mL |
| Model + low pH | 3.96 ±  0.22mL | 5.17 ±  0.73mL | 7.05 ±  1.02mL | 6.13 ±  0.83mL | 5.83 ±  0.71mL | 6.06 ±  0.67mL | 6.35 ±  0.46mL | 5.73 ±  0.87mL |
| Model + high pH | 4.90 ±  0.38mL | 6.71 ±  0.87mL | 6.00 ±  1.14mL | 6.02 ±  0.63mL | 5.84 ±  0.49mL | 6.84 ±  0.94mL | 6.18 ±  0.62mL | 7.81 ±  1.76mL |


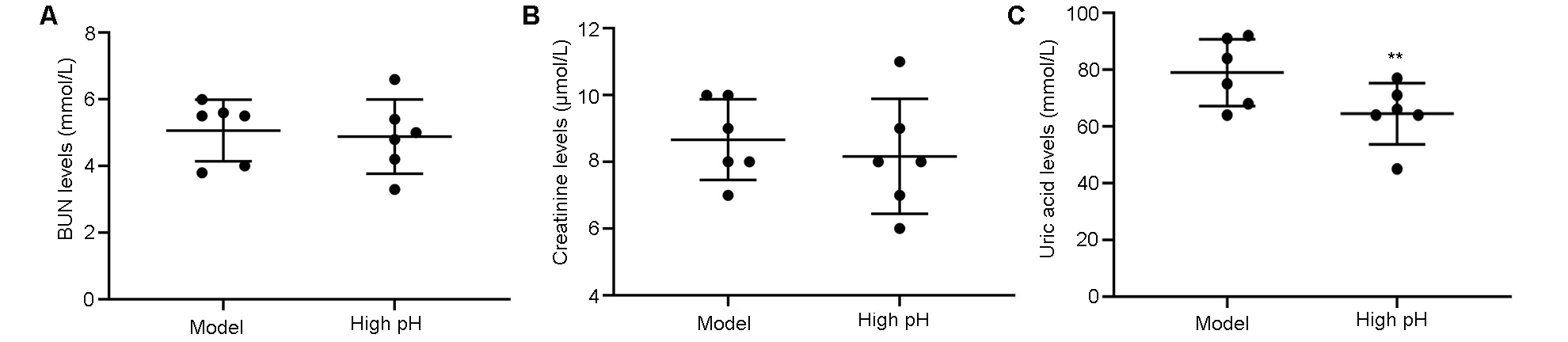


Figure S1: The serum BUN (A), creatinine (B), and Uric acid (C) levels in model group and high pH group.
